# Turbulent Drag Reduction with an Ultra-High-Molecular-Weight Water-Soluble Polymer in Slick-Water Hydrofracking

**DOI:** 10.3390/molecules27020351

**Published:** 2022-01-06

**Authors:** Juanming Wei, Wenfeng Jia, Luo Zuo, Hao Chen, Yujun Feng

**Affiliations:** 1 SINOPEC Research Institute of Petroleum Engineering, Beijing 100101, China; weijm.sripe@sinopec.com (J.W.); jiawf.sripe@sinopec.com (W.J.); zuoluo.sripe@sinopec.com (L.Z.); 2 State Key Laboratory of Polymer Materials Engineering, Polymer Research Institute, Sichuan University, Chengdu 610065, China

**Keywords:** water-soluble polymer, drag reducer, slick-water, hydrofracking, rheological behavior

## Abstract

Water-soluble polymers as drag reducers have been widely utilized in slick-water for fracturing shale oil and gas reservoirs. However, the low viscosity characteristics, high operating costs, and freshwater consumption of conventional friction reducers limit their practical use in deeper oil and gas reservoirs. Therefore, a high viscosity water-soluble friction reducer (HVFR), poly-(acrylamide-*co*-acrylic acid-*co*-2-acrylamido-2-methylpropanesulphonic acid), was synthesized via free radical polymerization in aqueous solution. The molecular weight, solubility, rheological behavior, and drag reduction performance of HVFR were thoroughly investigated. The results showed that the viscosity-average molecular weight of HVFR is 23.2 × 10^6^ g⋅mol^−1^. The HVFR powder could be quickly dissolved in water within 240 s under 700 rpm. The storage modulus (*G*′) and loss modulus (*G*″) as well as viscosity of the solutions increased with an increase in polymer concentration. At a concentration of 1700 mg⋅L^−1^, HVFR solution shows 67% viscosity retention rate after heating from 30 to 90 °C, and the viscosity retention rate of HVFR solution when increasing *C*_NaCl_ to 21,000 mg⋅L^−1^ is 66%. HVFR exhibits significant drag reduction performance for both low viscosity and high viscosity. A maximum drag reduction of 80.2% is attained from HVFR at 400 mg⋅L^−^^1^ with 5.0 mPa⋅s, and drag reduction of HVFR is 75.1% at 1700 mg⋅L^−^^1^ with 30.2 mPa⋅s. These findings not only indicate the prospective use of HVFR in slick-water hydrofracking, but also shed light on the design of novel friction reducers utilized in the oil and gas industry.

## 1. Introduction

Unconventional hydrocarbon resources, such as shale oil and gas, have garnered more attention and are being considered as an alternative energy resource to conventional oil and gas [1,2]. Shale oil and gas reservoirs require additional treatments, i.e., volumetric hydraulic fracturing, to achieve commercial production from tight formations due to their ultra-low permeability and ultra-low porosity [3,4,5]. In the volumetric hydraulic fracturing operation, higher pumping rates result in tremendous flowing frictional drag [6,7]. For this reason, the primary goal of slick-water is to reduce the frictional drag developed in pipeline flows [8,9]. Nevertheless, several challenges remain, including the usage of freshwater [10], high operation costs [11], environmental issues [12], and proppant transporting capability [6,11], which limit the applications of slick-water in the deeper (>3500 m) shale gas reservoirs. Therefore, it is significant and profound to develop an applicable water-soluble friction reducer for slick-water hydrofracking.

Normally, natural polysaccharides, such as xanthan gum [13,14], diutan gum [15] and guar gum [16], have been widely used as friction reducers since they are commercially available, environmentally acceptable and biologically degradable. Habibpour and Clark [13] investigated the drag reduction of xanthan gum, and found that the xanthan gum reduced frictional drag by 38% at concentrations of 600 mg⋅L^−^^1^. Soares and coworkers [15] examined the drag reduction properties of diutan gum and found a drag reduction of 24% to 70% at concentrations ranging from 50 to 800 mg⋅L^−^^1^ was attained. Habibpour et al. [16] studied the drag reduction behavior of guar gum at various concentrations. They discovered that the drag reduction increased from 5% to 18% as the concentration varied from 500 to 2000 mg⋅L^−^^1^. Nevertheless, the drag reduction performance is strongly related to the flexibility of polymers. Natural polysaccharide, as a typical rigid polymer, possesses a reasonably high drag reduction only when the concentration exceeds the critical overlap concentration, which implies that much higher operation costs are needed for slick-water hydrofracking. Besides, another major concerns with biopolymers is formation damage. Either insoluble residues of natural polysaccharide or plentiful solid residues after gel-breaking will inevitably block the pore throat of formation permeability [17,18]. Consequently, the utility of natural polysaccharides as friction reducers in slick-water is severely limited.

Owing to their high water solubility, reactive pendant groups, and low cost, polyacrylamide (PAM) and its derivatives are the most extensively employed friction reducers for slick-water hydrofracking [13,19,20,21]. The drag reduction performance of partially hydrolyzed polyacrylamide (HPAM) in a closed flow loop was investigated by Habibpour et al. [13]. A maximum drag reduction of 67% was obtained for the 1000 mg⋅L^−^^1^ HPAM solution. Feng et al. [22] have reported the drag reduction behavior of partially hydrolyzed polyacrylamide (HPAM) emulsion at a concentration of 300 mg⋅L^−^^1^ under high-temperature conditions. The HPAM emulsion demonstrated remarkable drag reduction performance, ranging from 77.5% to 70.2% when increasing the temperature from 35 to 65 °C, with a viscosity drop from 3.8 to 2.1 mPa⋅s. Bismarck et al. [23] measured the drag reduction of poly(acrylamide-*co*-sodium 2-acrylamido-2-methylpropanesulphonic acid) (poly(AM-*co*-AMPS)) under simulated field conditions. The drag reduction of such a water-soluble polymer increased from 34% to 64% when the polymer concentration was increased from 10 to 100 mg⋅L^−^^1^ with a viscosity elevated from 1.0 to 1.2 mPa⋅s. Jing et al. [24] reported an associative polymer friction reducer containing a nonionic hydrophobic monomer. The highest drag reduction of 70% was obtained when at polymer concentration of 500 mg⋅L^−^^1^ with a kinematic viscosity of 1.29 mm^2^⋅s^−^^1^ in brine containing 3.0 × 10^4^ mg⋅L^−^^1^ NaCl. However, due to its low viscosity characteristics, slick-water based on the aforementioned friction reducers has been restricted to suspending and/or transporting proppant. Therefore, slick-water hydrofracking must be consumed for immense quantities of water to deploy vast masses of proppant at low pumping concentrations, leading to high operation costs and environmental concerns accordingly.

The deficiencies of conventional friction reducers inspired the ingenious concept of high viscosity water-soluble friction reducers (HVFRs), which could be one of the most intriguing alternatives to friction reducers for slick-water hydrofracking [25,26,27]. Such types of polymers are acrylamide-based water-soluble copolymers with an ultra-high molecular weight (>10 × 10^6^ g⋅mol^−1^) which exhibit strong thickening power and prominent elasticity. Therefore, HVFRs greatly reduce the dosage of chemicals consumed and the volume of water required. Besides, HVFRs contain negligible residues compared to natural polysaccharides, implying that the formation permeability may be successfully recovered with breakers. Consequently, HVFRs have become widespread used in numerous basins around North America [11]. Nevertheless, to the best of our knowledge, few reports of HVFRs have been reported yet dealing with drag reduction. Moreover, slick-water exhibits better drag reduction performance at low viscosity as compared to its high viscosity counterparts.

In this work, we expect to obtain a HVFR with significant drag reduction performance for both low and high viscosities. Specifically, an HVFR was prepared by copolymerization of acrylamide, acrylic acid and 2-acrylamido-2-methylpropanesulphonic acid. The structure of HFVR was characterized through NMR spectroscopy and Fourier transform infrared spectroscopy (FTIR), respectively. Then the intrinsic viscosity of HFVR was measured to calculate its molecular weight. The solubility and rheological behavior of HVFR were examined using a rotational rheometer. Finally, the capacity of the HVFR to reduce frictional drag in slick-water hydrofracking was demonstrated with a closed-loop pipe system.

## 2. Results and Discussion

### 2.1. Structural Characterization

**Figure 1:**
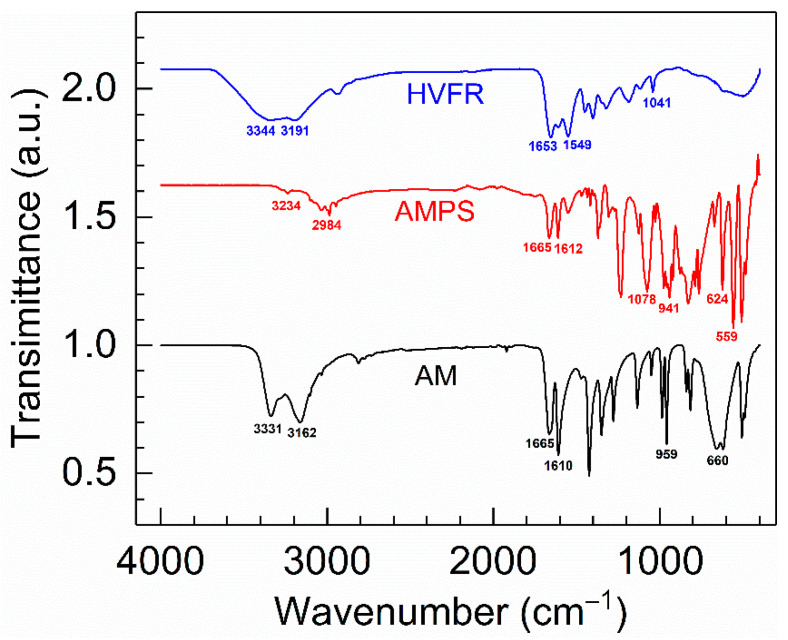
Comparison of the FTIR spectra of copolymer HVFR versus monomers AM and AMPS.

In order to validate whether the polymerization was successfully implemented, FT-IR was used to qualitatively characterize the structure of HVFR. Figure 1 gives the FT-IR spectra of the HVFR, AM and AMPS. Moreover, for better comparison, the transmittance of AMPS and HVFR increased by a multiple of 1.62 and 2.07 in Figure 1, respectively. The absorption bands at 3331, 3162, and 1610 cm^−1^ are ascribed to the N–H bond in AM. The absorption peak at 1665 cm^−1^ is assigned to the C=O bond in AM, and the peaks at 959 and 660 cm^−1^ corresponded to the C=C bond in AM. It is significantly different from AM, and the peaks at 1078 and 624 cm^−1^ are attributed to the −SO_3_^−^ in AMPS. As for HVFR, the N-H bond in –CONH_2_ was indicated by the absorption bands at 3344, 3191, and 1549 cm^−1^, and the characteristic absorption peak at 1653 cm^−1^ is relative to the C=O bond. The typical band at 1041 cm^−1^ is attributed to the −SO_3_^−^. Furthermore, the absorption characteristic peak of the C=C bond was not discovered, indicative of full conversion of the monomers into a polymer.

**Figure 2:**
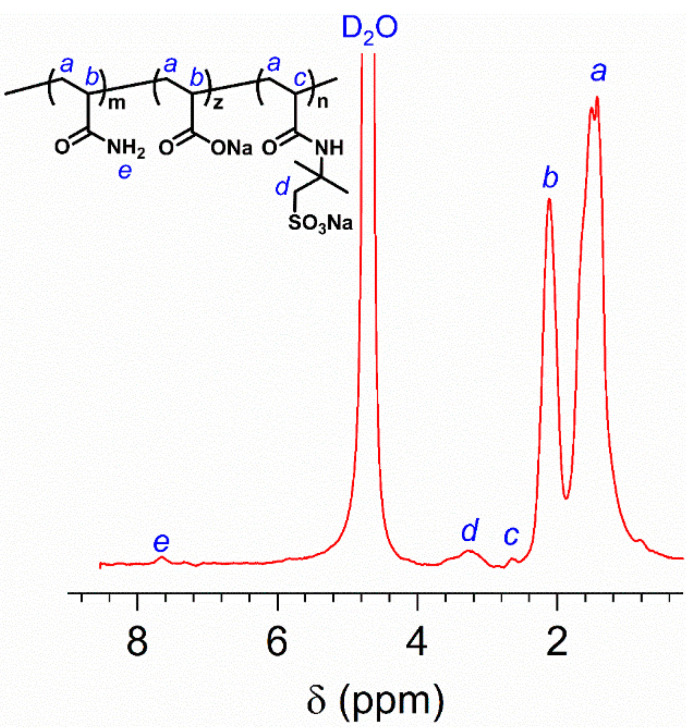
^1^H NMR spectrum of HVFR dissolved in D_2_O.

Moreover, the chemical structure of HVFR was also confirmed by ^1^H NMR spectroscopy. As depicted in Figure 2, the typical peak at chemical shift about 1.4 ppm (peak a) for –C***H***_2_– in polymer mainchain is evidenced. The proton peak at 2.1 ppm (peak b) for –C***H***– is related to the AM and AA. At 2.6 ppm (peak c), the proton signal for–C***H***– in AMPS is observed. The proton peak d (–C***H***_2_–) bonded with the −SO_3_^−^ is found at 3.3 ppm. The 4.8 ppm is peak of the solvent D_2_O. Thus, these results further verified the successful preparation of HVFR.

**Figure 3:**
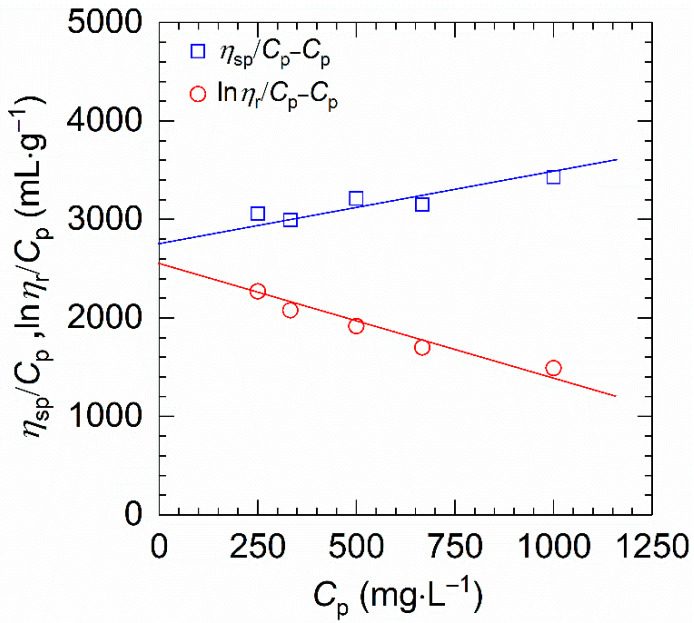
*η*_sp_/*C*_p_ and (ln*η*_r_/*C*_p_) plotted against polymer concentration for HVFR in 1 M NaCl solution at 30 °C.

On the other hand, owing to the high viscosity and ionic groups, the common methods such as gel permeation chromatography and static light scattering are not appropriate for measuring the molecular weight of HVFR [28]. Thus, we used the conventional viscometry to determine the molecular weight. Figure 3 exhibits the reduced viscosity (*η*_sp_/*C*_p_) and inherent viscosity (ln*η*_r_/*C*_p_) against polymer concentration (*C*_p_). One can find that both *η*_sp_/*C*_p_ and ln*η*_r_/*C*_p_ present a good linear dependence on the polymer concentration, indicating better fittings of the two equations and an accurate determination of [*η*]. The value of [*η*] for HVFR obtained by averaging the intersections of both plots is 2710 mL⋅g^−1^. Therefore, the viscosity-average molecular weight (*M*_η_) of the HVFR calculated from [*η*] through Mark−Houwink relationship is 23.2 × 10^6^ g⋅mol^−1^. Moreover, the resulting [*η*] can be used to suggest the overlap concentration, C*, which is defined as C* ≈ 1/[*η*]. In this work, the overlap concentration investigated is calculated to be 369 mg⋅L^−1^ for HVFR.

### 2.2. Solubility

Normally, the primary criterion for judging whether a polymer can be used as a drag reducer is its water solubility [17]. Thus, the dissolution time of HVFR was measured at an MCR 302 rheometer using a concentric cylinder geometry CC27 at a constant temperature of 30 °C and a fixed polymer concentration of 1000 mg⋅L^−1^ under different rotational speeds.

**Figure 4:**
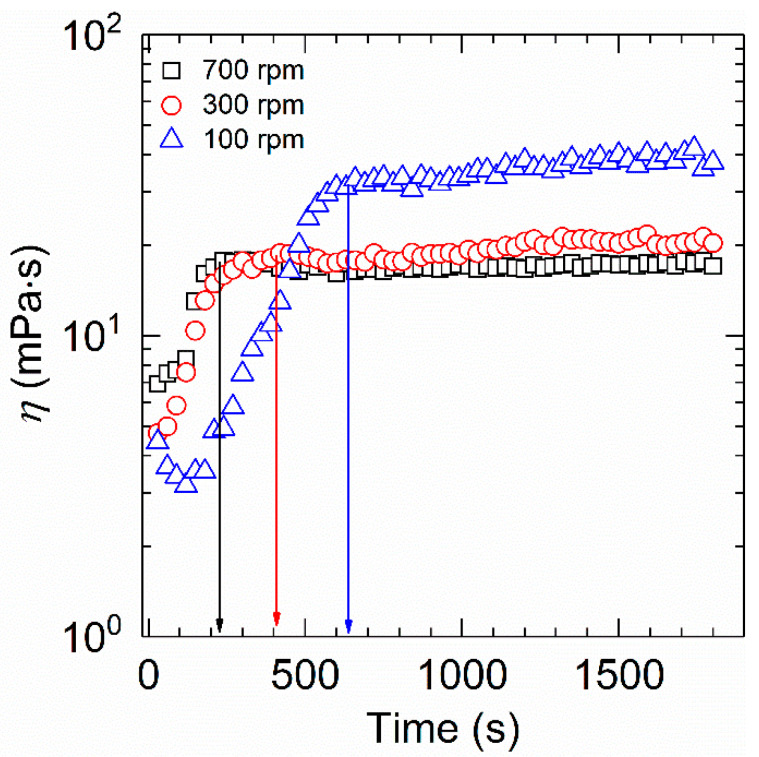
Determination of dissolution time of 1000 mg⋅L^−^^1^ HVFR at 30 °C.

The viscosity−time dependence for the aqueous solution of HVFR under different rotational speeds is presented in Figure 4. The shear viscosity in each curve sharply ascends and thereafter remains stable with time, indicative of full dissolution of the HVFR. Besides, it is clear that the dissolution time of HVFR increases from 240 to 630 s as the rotational speed decreases from 700 to 100 rpm. This could be ascribed to the fact that higher rotational speed induces strong violent turbulence, substantially improving the surface hydration of polymer powder and leading to reduction of the mass transfer resistance between the polymer powder and water. Therefore, the dissolution time of HVFR accordingly decreases. To the best of our knowledge, the dissolution time of HVFR is superior to that of the reported drag reducers [6,29].

### 2.3. Rheological Behaviors

#### 2.3.1. Steady Rheological Behavior

Generally, fracturing fluids suffer different shear rates during the fracturing process. For example, a higher shear rate in excess of 3880 s^−1^ is observed in shale fracturing treatments [30]. In contrast, a relative low shear rate inside the fractures is ranging from 10 to 100 s^−1^ [31]. Thus, the influence of shear rate on rheological properties of samples with different concentrations at a constant temperature of 30 °C in a shear rate scanning from 10^−3^ to 10^3^ s^−1^ was monitored.

**Figure 5:**
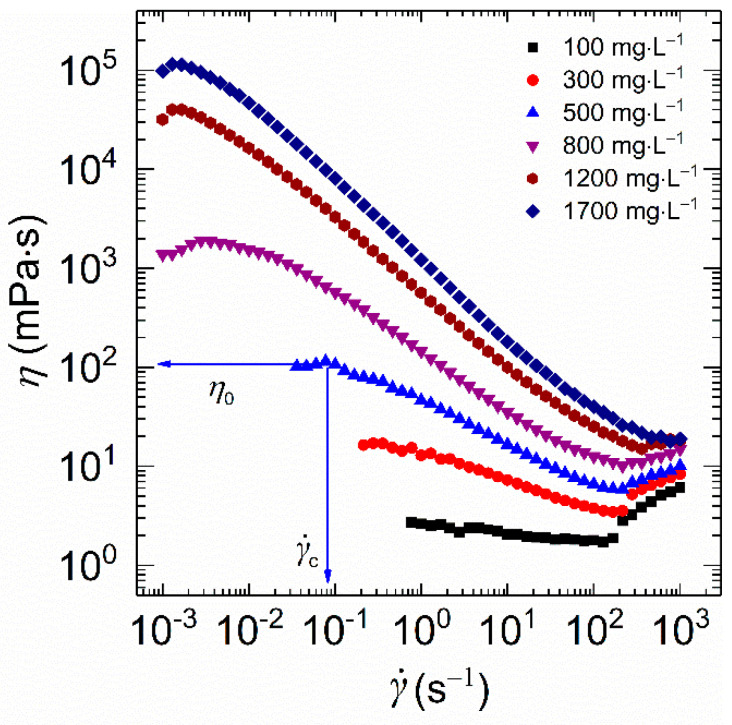
Shear viscosity plotted as a function of shear rate for HVFR solutions with different concentrations at 30 °C.

As described in Figure 5, within the concentration range studied, each curve exhibits Newtonian behavior at low shear rates with constant viscosity. Here, the constant value is referred to as the zero-shear viscosity (*η*_0_). Additionally, the high value of *η*_0_ indicates the presence of more entangled macromolecule chains. As for HVFR, the value of *η*_0_ presented a monotonic increase with polymer concentration. The value of *η*_0_ for 1700 mg⋅L^−1^ HVFR polymer solution is several orders of magnitude higher than its low polymer concentration solution counterparts, implying the presence of more entangled networks of polymer chains in the concentrated solution. Moreover, the value of *η*_0_ for 1700 mg⋅L^−1^ HVFR polymer solution (11.5 × 10^4^ mPa⋅s) is better than the reported 6000 mg⋅L^−1^ HVFR-900 solution [27], indicating a remarkable thickening ability of HVFR. The onset of shear-thinning appears at a critical shear rate (${\dot{\gamma }}_{c}$), in which viscosity decreases with an increase in shear rate. The minimal value ${\dot{\gamma }}_{c}$ is found for concentrated polymer solutions, suggesting its more significant shear-thinning response. Such shear-thinning behavior is a representative of non-Newtonian fluids. This behavior furnishes the polymer with improved flowing ability during the hydrofracturing process. In other words, the HVFR polymer solutions can be easily pumped into the target area. Moreover, due to the Taylor instabilities [32,33], a shear-thickening behavior was observed at high shear rates (>100 s^−1^).

Generally, fracturing fluids are mainly used in high-temperature and high-salinity (HTHS) environment [34,35]. Therefore, it is necessary to further investigate the salinity tolerance and thermal stability of HVFR polymer solution.

**Figure 6:**
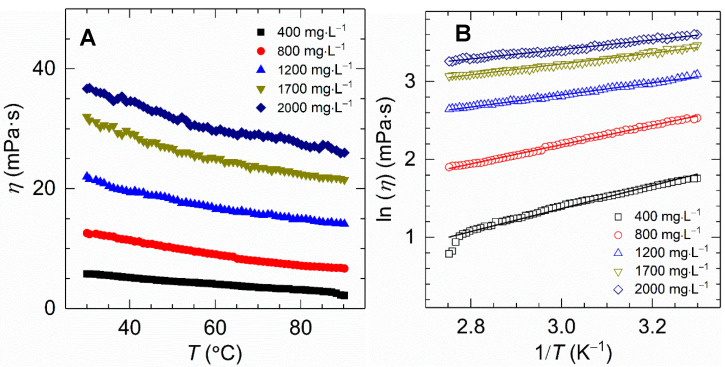
(**A**) Effect of temperature on apparent viscosity for HVFR solutions with different concentrations; (**B**) Logarithmic plots of viscosity against the reciprocal of temperature for HVFR.

All experiments were subjected to temperature scanning from 30 to 90 °C with a constant heating rate of 2 °C⋅min^−1^ and a fixed shear rate of 170 s^−1^. Figure 6A displays the viscosity variation of the HVFR solution versus temperature for polymer concentrations varying from 400 to 2000 mg⋅L^−1^. A thermothinning behavior was found for all samples and the changing trend as most normal polymers do [36]. For example, the viscosity of 1700 mg⋅L^−1^ HVFR polymer solution reduced from 32 to 21 mPa⋅s when temperature was increased from 30 to 90 °C. The reduction of solution viscosity as a result of elevated temperature intensifying thermal motion of water molecules and polymer chains, as well as deteriorating intermolecular action within polymer chains [7]. As to our knowledge, the viscosity of previously reported HVFR with concentration of 4000 mg⋅L^−1^ can be maintained at 16 mPa⋅s under 75 °C and 100 s^−1^ [11], which is obviously lower than HVFR at 75 °C and 170 s^−1^ in this work.

Furthermore, the temperature effect of polymer solution viscosity (*η*) can be correlated to the Arrhenius equation:

(1) $$\eta =A\exp\;(\frac{E_{a}}{\mathrm{RT}})$$

Double-logarithm of Equation (1) gives,

(2) $$\ln\eta =\frac{E_{a}}{\mathrm{RT}}+\ln A$$

where *A* is the frequency factor, *E*_a_ refers to the activation energy, *R* represents the gas constant in kJ⋅mol^−1^⋅K^−1^, and *T* denotes the absolute temperature in K.

As shown in Figure 6B, a linear relationship between the ln*η* and 1/*T* is observed for all HVFR solutions, suggesting the viscosity of HVFR decreases with the increasing temperature generally following the Arrhenius law.

**Figure 7:**
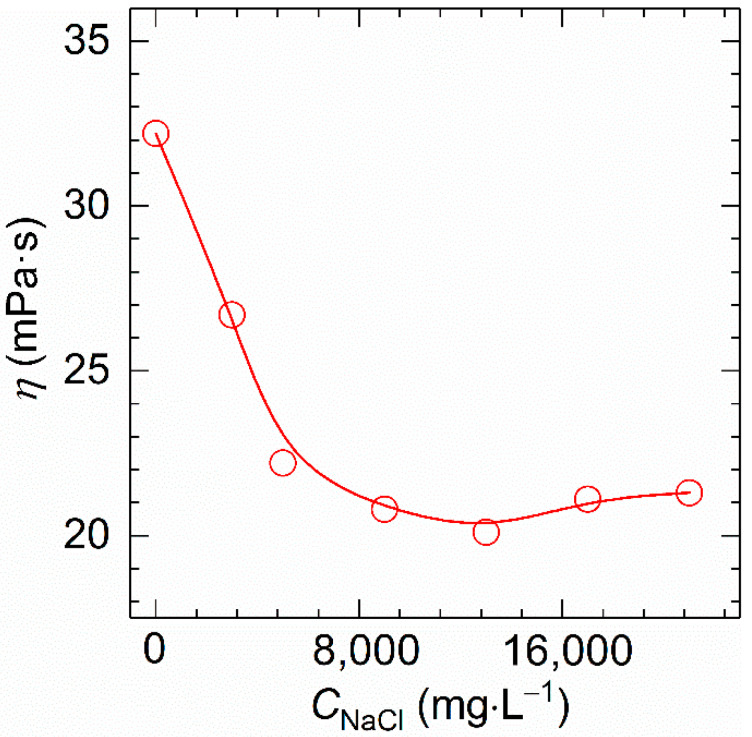
Variation of the apparent viscosity versus NaCl concentration for 1700 mg⋅L^−^^1^ HVFR at 30 °C.

Next, the viscosity of polymer solution with 1700 mg⋅L^−^^1^ polymer concentration was observed at 30 °C and 170 s^−^^1^ at different NaCl concentrations ranging from 0 to 2.1 × 10^4^ mg⋅L^−^^1^. As shown in Figure 7, the viscosity of HVFR stays at a high level while changing sharply when increasing the *C*_NaCl_ to 13,000 mg⋅L^−1^, and a slight augmentation in viscosity was seen with a further increase of the *C*_NaCl_ to 21,000 mg⋅L^−1^. Since HVFR contains carboxylate groups, it behaves as a typical anionic polyelectrolyte, very sensitive to the presence of cations. Consequently, the viscosity of HVFR solution would be decreased owing to the screening of the electrostatic repulsions among the polymer chains. The explanation for salt-induced viscosifying behavior is connected to the fact that ions shield the repulsive intermolecular interactions to facilitate the formation of hydrogen bonds between the compact polymer chains [17,37]. In summary, at concentration of 1700 mg⋅L^−1^, HVFR solution shows 67% viscosity retention rate after heating from 30 to 90 °C, and the viscosity retention rate of HVFR solution when increasing *C*_NaCl_ to 21,000 mg⋅L^−1^ is 66%. Consequently, HVFR exhibits acceptable thermal stability and salt tolerance.

#### 2.3.2. Dynamic Rheological Behavior

To the best of our knowledge, the elastic properties of fracturing fluids are crucial to their proppant-carrying capacity [38], which is also related to the dynamic rheological response of HVFR solution here. In this section, we carried out delicate dynamic rheological measurements, i.e., strain sweep and frequency sweep tests, at 30 °C.

**Figure 8:**
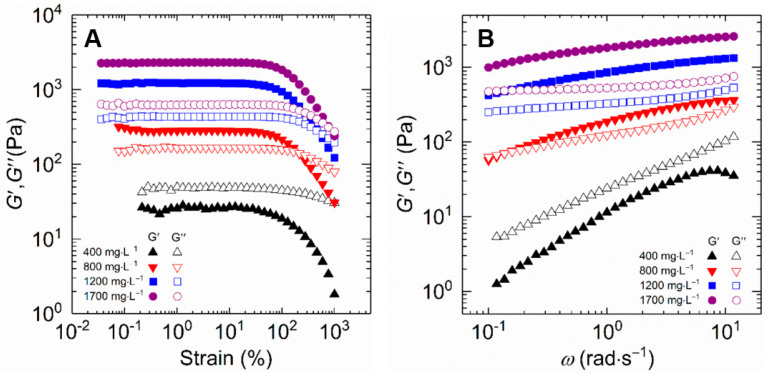
The storage and loss modulus plotted as a function of (**A**) strain and (**B**) frequency for HVFR solution at 30 °C.

Figure 8A compares the viscoelastic characteristics of HVFR solution from strain sweep experiment with polymer concentration altering from 400 to 1700 mg⋅L^−1^ at 30 °C and an angular frequency (*ω*) of 6.28 rad⋅s^−1^, with strain ranging from 0.01 to 100%. The HVFR solutions exhibit the same shape for storage modulus (*G*′) and loss modulus (*G*″) on oscillatory strain (*γ*) at all polymer concentrations. At 0.01% < *γ* < 100%, the *G*′ and *G*″ independent of *γ* are observed, which reflects a linear viscoelastic region. The constant values of *G*′ and *G*″ within this linear viscoelastic region indicate that the network structure of HVFR is intact and changes inconspicuously with varied *γ*. When *γ* is above 100%, both *G*′ and *G*″ gradually decrease with *γ*, implying the disentanglement and orientation of polymer chains of HVFR at high *γ*. Besides, as HVFR concentration increases, both *G*′ and *G*″ accordingly increase, demonstrating concentrated HVFR solutions exhibit more significant viscoelasticity than its low polymer concentration counterparts. Nevertheless, the values of *G*′ at HVFR concentration ranging from 800 to 1700 mg⋅L^−1^ are always higher than the *G*″ in the linear viscoelastic region, implying that these HVFR polymer solutions have elastic dominated behavior. Conversely, the *G*″ of 400 mg⋅L^−1^ HVFR polymer solution is greater than the *G*′, which shows the viscous predominate behavior. Here the polymer solutions can be separated into dilute and semi-dilute domains by overlapping concentration 369 mg⋅L^−1^ as mentioned above. In this respect, all employed HVFR concentrations are above the overlap concentration, falling in the semi-dilute regime. At polymer concentration of 400 mg⋅L^−1^, although entanglement between the polymer chains occurs, whereas the intermolecular interactions between the macromolecule chains are relative weak. These interactions are not sufficient to improve the viscoelasticity of the polymer solution, which is therefore predominantly viscous. With increasing polymer concentration, there is a notable increase in intermolecular interactions and entanglements, resulting in the viscoelasticity of polymer solution transition from a predominantly viscous to an elastic response.

Furthermore, the results of *G*′ and *G*″ from frequency (*ω*) sweep tests were measured in the linear viscoelastic region at a fixed temperature of 30 °C, with frequency varied from 10^−1^ to 10 rad⋅s^−1^. Figure 8B compares the frequency sweep spectra of HVFR polymer solution at various polymer concentrations. Both *G*′ and *G*″ increase with the increase in polymer concentration. In addition, it is found that the 800 mg⋅L^−1^ HVFR solution shows viscoelastic response behavior, that is to say, a viscous response (*G*′ < *G*″) at low *ω* while an elastic behavior (*G*′ > *G*″) at high *ω*. The polymer solutions with polymer concentration above and below 800 mg⋅L^−1^ exhibit viscous behavior (*G*′ < *G*″) and gel-like response (*G*′ > *G*″) over the frequency range, respectively.

### 2.4. Drag Reduction Performance

Apart from the rheological properties, one of the most essential parameters targeting the application of fracturing fluid in slick-water hydrofracking for shale gas is drag reduction performance. Hence, the performance of HVFR solutions was investigated at various HVFR concentrations and fluxes.

**Figure 9:**
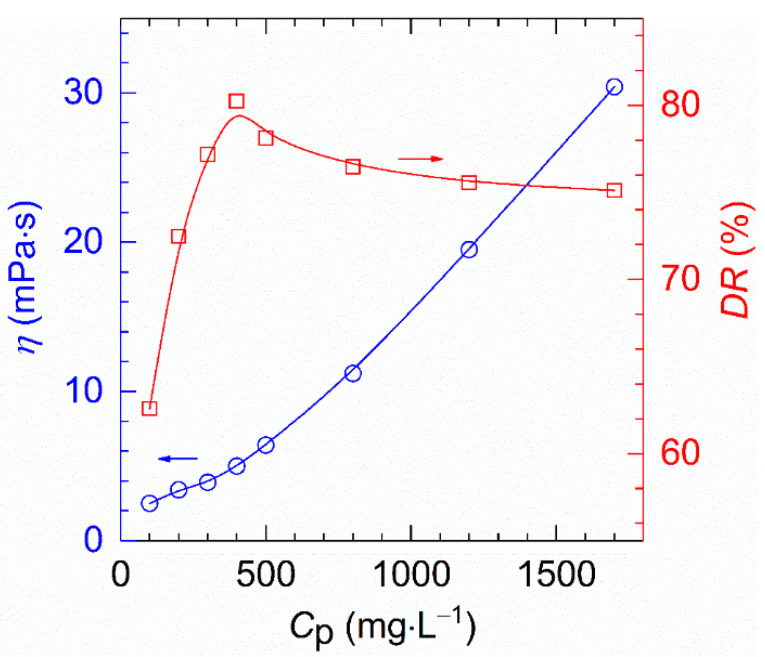
The dependence of drag reduction and apparent viscosity on polymer concentration (T, 30 °C; shear rate, 170 s^−1^; flux, 170 L⋅min^−1^).

The drag reduction of HVFR polymer solutions show a single peak curve (see Figure 9), which was similar to the results reported by Zhang et al. [39]. While the viscosity of HVFR samples increases monotonously with polymer concentration, the drag reduction initially arise from 62.6% to 80.2% when polymer concentration increases from 100 to 400 mg⋅L^−1^, and afterwards gently decreases from 80.2 to 75.1% when polymer concentration reaches up to 1700 mg⋅L^−1^. This finding can be ascribed to the fact that, before the polymer concentration of 400 mg⋅L^−1^, the increased viscosity dampens the formation and growth of eddies and thus intensifying the drag reduction [6,22]. Beyond the concentration of 400 mg⋅L^−1^, the increase in polymer concentration leads to a sharp increase in viscous as well as viscous resistance and thus deteriorating of drag reduction ability [22,23]. Therefore, the dependence of polymer concentration terminates when the polymer concentration exceeds this point. Generally, the polymer concentration where maximum drag reduction occurs is known as the optimum concentration [40]. That is to say, the concentration of 400 mg⋅L^−1^ is the optimum concentration for HVFR. Remarkably, to the best of our knowledge, the HVFR exhibited better drag reduction performance than previously-reported HVFR-900 with the same viscosity [27].

**Figure 10:**
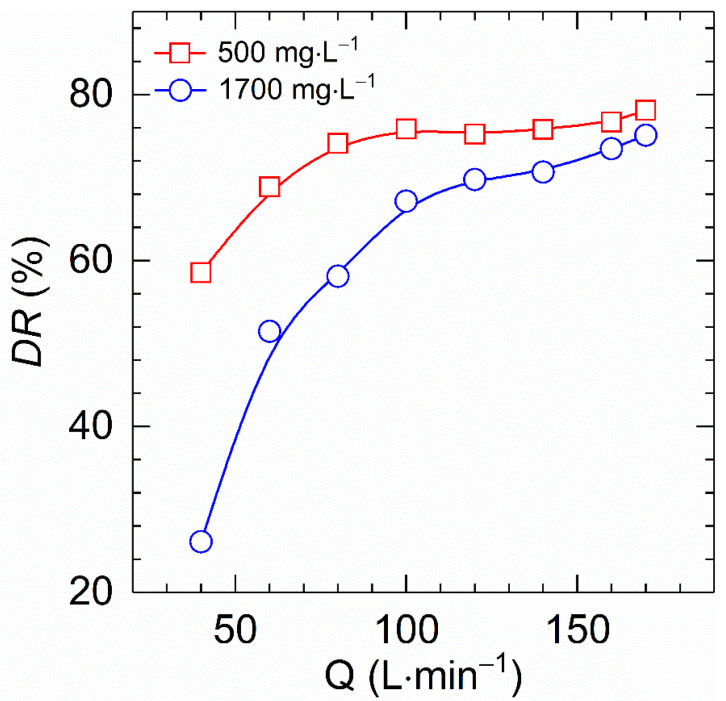
Comparison of the drag reduction versus flux for HVFR at 500 and 1700 mg⋅L^−1^ at 30 °C.

Figure 10 describes the dependence of drag reduction of HVFR solutions with polymer concentration of 500 and 1700 mg⋅L^−1^ on fluxes at 30 °C. All curves show a monotonic increase in drag reduction with increasing flux, which is in good agreement with reported result of drag reducers [6,22,27]. The explanation for these results is that drag reduction performance is linked to turbulence intensity, which is determined by the Reynolds number. On the other hand, the Reynolds number is solely governed by the flow rate for a given polymer solution and test conditions. As a result, the elevated flux reinforces turbulence intensity and thereby improving the drag reduction ability. Moreover, the drag reduction was found to be more dependent on the flux for high polymer concentration solutions than for low polymer concentration solutions. As the fluxes increased from 40 to 170 L⋅min^−1^, the drag reduction of the HVFR solution at 500 mg⋅L^−1^ concentration improved 58.5 to 78.1% while enhanced from 26.1 to 75.1% for 1700 mg⋅L^−1^ HVFR solution. In short, it can be concluded that HVFR solutions with low and high polymer concentrations have significant drag reduction performances.

## 3. Materials and Methods

### 3.1. Materials

Acrylamide (AM), 2-Acrylamido-2-methylpropanesulphonic acid (AMPS), 2,2′-Azobis [2-(2-imidazolin-2-yl) propane] dihydrochloride (V044), were purchased from Sigma-Aldrich (Burlington, MA, USA) and used without further treatment. Ammonium persulfate [(NH_4_)_2_S_2_O_8_], sodium hydrogen sulfite (NaHSO_3_), sodium hydroxide (NaOH) and sodium chloride (NaCl) of analytical grade were obtained from Chron Chemicals (Chengdu, China). The Deionized water with a resistivity of 18.25 MΩ⋅cm was prepared using a CDUPT-III ultrapure water purification system (Chengdu Ultrapure Technology Co., Ltd., Chengdu, China).

### 3.2. Synthesis

**Scheme 1:**
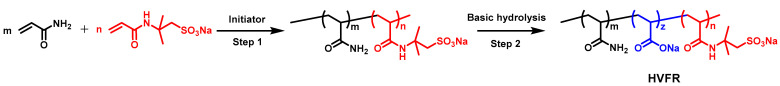
Schematic route toward the preparation of HVFR.

The HVFR was prepared by free radical polymerization in deionized water in an oxygen-free nitrogen atmosphere. The appropriate amounts of AM (95 g) and AMPS (5 g) were dissolved in deionized water (400 g) and the pH of the solution was adjusted to 7~8 by NaOH. Afterwards, the solution was transferred into a thermostatic container and the solution temperature was cooled to 10 °C under a continuous flow of nitrogen. Then the initiators including V044 (0.01 g), NaHSO_3_ (0.01 g), and (NH_4_)_2_S_2_O_8_ (0.01 g) dissolved in deionized water were slowly added into aforementioned solution. The polymerization was kept for 24 h to ensure a full reaction. After polymerization, the polymer was first added into a deionized water while stirring for about 5 h to ensure fully dissolution. Then, appropriate amounts of NaOH was dissolved into polymer solution. Next, the solution was heated to 80 °C and kept for 2 h. The final product was dried at 40 °C first, then dissolved in deionized water, followed by precipitation several times in excess of ethanol and was freeze-dried to obtain the final white powder. The reaction route is illustrated in Scheme 1.

### 3.3. Structural Analysis

The molecular structure of HVFR was determined by ^1^H NMR and Fourier transform infrared (FTIR) spectra, respectively. An AVANCE 800 MHz spectrometer (Bruker, Karlsruhe, Germany) was used for ^1^H NMR with D_2_O as the solvent, where the spectra were recorded under room temperature. FTIR spectroscopy measurement was carried out with NEXUS 380 FTIR spectrometer (Nicolet, Madison, WI, USA) in the 500–4000 cm^−1^ with a resolution of 4 cm^−1^ at room temperature.

### 3.4. Determination of Molecular Weight

1.0 M NaCl aqueous solution and five polymer aqueous solutions with different concentrations prepared by same solvent (1.0 M NaCl) were placed in an IVS400 automatic capillary viscometer (Zonwon Technology Co., Ltd., Hangzhou, China) with a capillary inner diameter of 0.55 mm at 30 °C. The running time of polymer solutions (*t*_1_) and the solvent (*t*_0_) was used to calculate the relative viscosity *η*_r_ = *t*_1_/*t*_0_, and specific viscosity *η*_sp_ = (*t*_1_ − *t*_0_)/*t*_0_ [28]. The intrinsic viscosity, [*η*], was obtained by extrapolating to infinite dilution and taking the intercept from Huggins and Kraemer diagrams. The viscosity-average molecular weight (*M*_η_) of the polymer can be determined from [*η*] via the Mark-Houwink equation:

(3) $$\left[\eta \right]={kM}_{\eta }{}^{\alpha }$$

The *k* and *α* refer to the parameters for Mark-Houwink equation. The value of *k* and *α* used in this paper is 3.73 × 10^−4^ and 0.66 respectively [37,41].

### 3.5. Rheological Test

All rheological measurements were performed on a Physical MCR 302 rotational rheometer (Anton Paar, Graz, Austria) with concentric cylinder geometry CC27. The measuring bob and cup have radii of 13.33 and 14.46 mm, respectively. During the steady rheological test, the value of viscosity was measured after a test time at each shear rate according to relationship $\dot{\gamma }$ × *t* ≥ 1. The strain sweep tests were used to determine the linear viscoelastic regime for the dynamic rheological investigations. A Peltier temperature control device was used to set the test temperature. In all experiments, samples were kept stable at test temperature for 5 min ahead of data collection.

### 3.6. Drag Reduction Measurement

The drag reduction was performed on a closed-loop pipe system (Jiangsu Huaan Scientific Research Instruments, Hai’an, China) equipped with pipe internal diameter of 16 mm and pipe total length of 4 m. The temperature inside the test section was held at 30 ± 0.5 °C by a temperature controller. Polymer powder was slowly sprinkled over a large solvent area under continuous stirring for 6 h, and the stirring rate was set at 200 rpm to avoid polymer mechanical degradation.

Once the polymer solutions were prepared, the screw pump was used to drive the polymer solutions and pure solvent flow in the closed-loop pipe system. The differential pressure transmitter and flow meter were separately used to measure the pressure drop and flux of the test section. Thereafter, the following Equation (4) was sued to calculate drag reduction at a given flow rate:

(4) $$DR=(1-\frac{P_{p}}{P_{s}})\;\times \;100$$

where *P*_s_ and *P*_p_ refer to the pressure drop measured in pure water and polymer aqueous solutions, respectively.

## 4. Conclusions

In this work, a high viscosity water-soluble friction reducer (HVFR) was synthesized by free radical polymerization in aqueous solution. The molecular weight, solubility, rheological properties, and drag reduction behavior of HVFR were systematically studied. The results showed that the viscosity-average molecular weight of HVFR is 23.2 × 10^6^ g/mol. The HVFR powder exhibited a rapid polymer dissolution rate. The basic rheological experiment results showed that HVFR has a remarkable thickening ability and viscoelastic behavior. The drag reduction test of the HVFR found that the HVFR showed significant drag reduction performance for both low viscosity and high viscosity. The drag reduction initially reaches its maximum drag reduction of 80.2% at 400 mg⋅L^−1^ with 5.0 mPa⋅s, and then slightly decreases to 75.1% when polymer concentration reaches up to 1700 mg⋅L^−1^ as well as viscosity to 30.2 mPa⋅s. The elevated flux reinforces turbulence intensity, resulting in improved the drag reduction performance. Additionally, the drag reduction was found to be more dependent on the flux for high polymer concentration solutions.

These findings not only provide a novel material for slick-water fracturing fluids but also open up a new avenues for synthetic polymers to shale oil and gas development industry in the future. Although the significant drag reduction performance of HVFR both for low viscosity and high viscosity, further investigation is needed before practical application can be realized, for instance, the proppant carrying capacity, gel breaking performance, and shear resistance ability.

## Data Availability

The data presented in this study are available on request from the corresponding author.

## Sample Availability

Samples of the HVFR is available from the authors.
